# *In Vivo* Bioavailability and Therapeutic Assessment of Host-Guest Inclusion Phenomena for the Hydrophobic Molecule Etodolac: Pharmacodynamic and Pharmacokinetic Evaluation

**DOI:** 10.3797/scipharm.0909-08

**Published:** 2010-01-18

**Authors:** Vivek Ranjan Sinha, Amita, Honey Goel

**Affiliations:** University Institute of Pharmaceutical Sciences, Panjab University,160014,Chandigarh, India

**Keywords:** ETD, β-Cyclodextrin, Inclusion-complexes, Bioavailability, Ulcerogenicity

## Abstract

The aim of present investigation was 1) to evaluate the in vivo bioavailability of an Etodolac (ETD)-β-cyclodextrin (β-CD) inclusion complex system prepared by kneading and spray drying techniques in rats, 2) to study the pharmacodynamic parameters in various animal models for analyzing the therapeutic response and, 3) to evaluate the pharmacokinetic profile of the drug administered. Inclusion complexation with β-CD enhanced the solubility of the drug, improved bioavailability and reduced ulcerogenicity of ETD in rats. Pharmacodynamic studies were carried out in normal LACA mice and pharmacokinetic evaluation was done in male Wistar rats. Pharmacokinetic parameters evaluated for the inclusion complexes revealed good correlation. The minimum dose necessary to produce analgesic or anti-arthritic activity was also decreased, indicating that the host-guest strategy that uses β-CD and ETD was very effective and could be successfully employed in the preparation of pharmaceutical formulations of anti-arthritics and analgesics.

## Introduction

ETD [(±)-1,8-diethyl-1,3,4,9-tetrahydropyrano[3,4-*b*]indol-1-yl)acetic acid] is an indole analog which acts as an analgesic (selective COX-2 inhibitor) and anti-inflammatory drug especially for the treatment of arthritis. The anti-inflammatory activity of ETD has been reported to be mainly due to the inhibition of prostaglandin biosynthesis [1]. This drug has a very poor solubility in water and this limits its use to solid dosage forms for oral administration. The major drawbacks associated with the NSAIDs are local gastrointestinal toxicity and ulceration. Upon chronic use, gastrolestivity lead to termination of NSAID therapy in such conditions. ETD, being a practically insoluble candidate, was evaluated for its toxicity and therapeutic assessment in the rats locally, as systemic side-effects were less prominent.

Recently, clinical studies have revealed that ETD chemically induced anticarcinogenic effects on various human carcinoma cell lines [2, 3] and have shown chemopreventive action on biliary carcinogenesis in hamsters [4]. Hence, ETD has revitalized the interest among the researchers for better management of NSAID therapies to widen the scope of therapeutic options for antiarthritis. Inclusion-complexation via cyclodextrins is one of the strategic tool for clinically useful candidates like ETD with poor preformulation characteristics. However, various investigations pertaining to complexation using ETD has been reported like solid dispersions [5–7].

In our previous work, we successfully encapsulated the hydrophobic molecule ETD into the CD moiety and enhanced various physiochemical factors like solubility behavior and dissolution rate [8]. Hence, the aim of the present study was to evaluate the in-vivo bioavailability and therapeutic assessment of ETD-β-CD complexes in male LACA mice or wistar rats. The pharmacodynamics of the inclusion complexes to study effect of toxicity on local tissues and pharmacokinetic profile to assess the bioavailability and peak plasma concentration was elucidated.

## Results and Discussion

### Pharmacodynamic studies

**Fig. 1.**
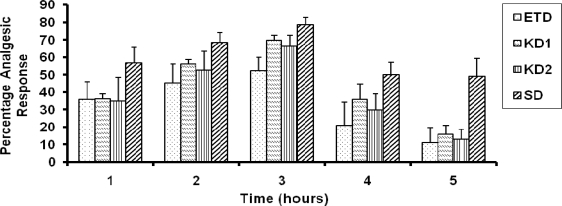
Percentage analgesic response of KD1, KD2, and SD as compared to the pure drug ETD (n=5, dose∼10mg/kg of drug).

The data of in vivo pharmacological responses (% analgesic effect) versus time for mice has been displayed in Fig.1. Initially the response was low due to slow absorption of the drug. A significant difference in the response of the 1:1 inclusion complexes (KD1) was observed at a time point of 1 h, 2 h and 4 h. It was highest after two hours of administration however after that there was a decline in the response. This may be attributed to decrease in the therapeutic level in the blood. However, KD2 exhibited a significant difference at 2 h and 4 h. The peak response was observed at 2 h in case of both the complexes as well as the pure drug. The response exhibited by the inclusion complexes, KD1 and KD2 was 1.33 and 1.27 fold higher respectively as compared to the pure drug at 2 h. Analgesic response exhibited by SD was significantly higher at all time points in comparison to pure drug, physical mixture and both the kneaded complexes i.e. KD1 and KD2. This data suggests that inclusion complexation was helpful in enhancing the analgesic response of the ETD.

### Acetic acid induced writhing for the determination of analgesic response of complexes over pure drug

**Fig. 2.**
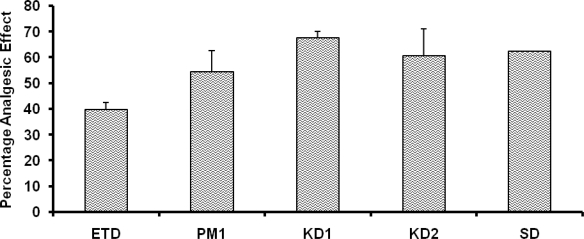
Percentage analgesic response of KD1, KD2, SD as compared to the pure ETD (n=5, dose∼10mg/kg of drug)

The percentage analgesic response for the inclusion complexes was significantly higher for the inclusion complexes when compared with the pure drug as well as the physical mixture (Fig. 2). This suggests that the inclusion complexes were more effective in preventing the acetic acid induced writhings. This can be attributed to the better absorption of the drug from the inclusion complexes as compared to the pure drug and the physical mixtures. The percentage analgesic response was 1.70 times higher in case of KD1 when compared with that of the pure drug. In case of KD2, it was 1.53 times higher in comparison of the pure drug. SD showed 1.56 times higher analgesic effect in comparison to pure drug. When compared with that of the physical mixture, KD1 and KD2 showed a 1.24 and 1.12 times which was significantly (p<0.05) higher analgesic response respectively. For SD, percent analgesic effect was 1.15 times higher than the physical mixture. These data suggests that inclusion complexation was helpful in enhancing the analgesic response of the ETD.

### Anti-inflammatory activity

**Fig. 3.**
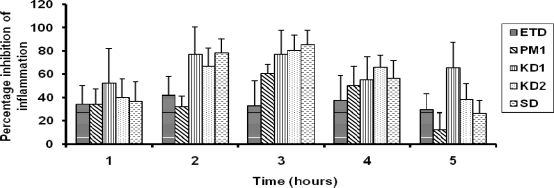
Percentage inhibition of inflammation of KD1, KD2, SD, ETD and the PM1(n=5, dose∼10mg/kg of drug).

The % anti-inflammatory activity for the inclusion complexes, KD1 and KD2 was significantly higher than that of pure drug, suggesting that the inclusion complexes were more effective in preventing the rat paw edema caused by the carrageenan (Fig. 3).

This can be attributed to the higher drug release from the inclusion complexes as compared to the pure drug and the physical mixtures. Significant difference (p<0.05) was observed at all the time intervals in case of KD1 complex except at 1 h and 4 h time interval, in comparison to the pure drug. KD2 complex also showed a significant difference from pure drug at 2 h, 3 h and 4 h time interval. The physical mixture showed a higher anti-inflammatory response which was significant only at 3 h in comparison to the pure drug. The lowest anti-inflammatory response was obtained by the pure drug. Highest response was observed at 3 h in case of both the kneaded complexes. The response with KD1 or KD2 was 2.35 times and 2.45 times higher than that of the pure drug at 3 h respectively. Physical mixture showed a response that was 1.85 times that of the pure drug at 3 h. These observations showed that inclusion complexes were more effective in inhibiting the anti-inflammatory response of carrageenan. SD complex also showed a significantly higher (p<0.05) anti-inflammatory response at 2 h and 3 h in comparison to both pure drug and physical mixture. In comparison to the physical mixture all the complexes showed higher anti-inflammatory response at different time points. KD1 showed a significant difference (p<0.05) at 1 h, 2 h and 6 h while KD2 showed a significant difference at 2 h, 3 h and 6 h in comparison to the physical mixture.

### Antiulcer activity

Presence of the undissolved particles of the drug in contact with the gastric mucosa leads to high local concentration of the drug. This high concentration leads to local irritation and finally causes ulceration of the mucosa. The complexation of the drug with β-CD, promotes the rapid dissolution of the drug. This promotion of dissolution reduces the presence of undissolved particles adhering to the gastric mucosa leading to the reduction in the ulcerative tendency of the drug. Minor ulceration produced in the inclusion complexes of the drug can be due to the systemic effect of the drug, resulting from the inhibition of the synthesis of prostaglandins PGI_1_ and PGE_2_, which have a protective action on the gastric mucosa. Based upon the ulcer index, the antiulcer activities for the various groups were classified on scale [9]. For the acute gastric damage, ETD produced significant augmentation in ulcer activity (with the ulcer index of 5.0) index as compared to control group and the complex treated one (Table 1). All the complexes showed better antiulcer activity, However, kneaded complexes KD1, KD2 exhibited better antiulcer activity as compared to spray dried complex (SD). On uclerogenic scale, SD showed a higher ulcer index (SD =1.5; KD1=1; KD2 =1). This may be attributed to the presence of some uncomplexed drug as supported by DSC studies [8].

### Pharmacokinetic studies

**Fig. 4.**
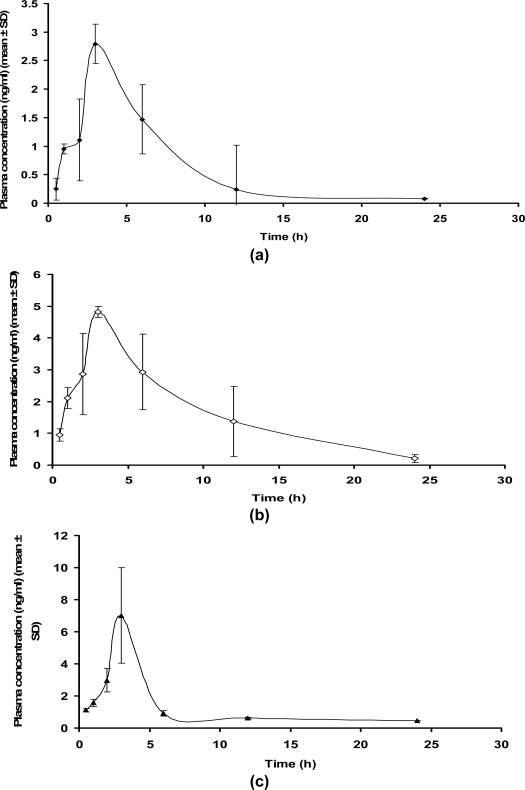
Mean plasma concentration (AUC_0–24h_) time profile of pure drug and complexes in wistar rats (n=5, dose eqv.∼ 10mg/kg of drug) a) ETD, b) KD1, c) SD

Pharmacokinetic evaluation of ETD (Fig. 4a) as well as the inclusion complexes was carried out in wistar male rats for 24 hours. Various pharmacokinetic parameters were calculated using PC-NONLIN software and were statistically compared using one way ANOVA followed by Tukey’s test (Table 2). The parameters were found to be significantly different (p<0.05) for other formulations in-vivo. For the pharmacokinetic profile of ETD, average max. concentration in plasma was found to be 2.79 ng/ml within 3 hrs after the oral administration of the drug. However, there was a decrease in the concentration suggesting fast elimination of ETD. The average elimination rate constants and AUC after the administration of pure drug was obtained to be 0.149 h^−1^ and 17.38 ngh/ml respectively.

### In vivo plasma studies using kneaded inclusion complexes

Pharmacokinetics of the complexes was performed in order to study its effect on the bioavailability of the drug and the data obtained was compared with that of pure drug. After administration of a single dose of 10 mg/kg, the plasma levels (AUC_0–24h_) were analyzed for the pure ETD over the next 24 h period. The mean serum concentration vs time profile for pure drug and KD1 has been depicted (Fig. 4b). Following the oral administration, the plasma levels increased sharply whereas C_max_ value reached within 3 h post dose. The average C_max_ value for the complex was 4.86 ng/ml, which was 1.74 times higher as compared to that observed with the pure drug. The t_max_ of the complex was almost similar to that of the pure drug but the extent of absorption was much higher in case of the complex as compared to the pure drug. It can be concluded from the values of AUC_t_∼43.84 ngh/ml that complexes exhibited greater absorption as compared with the pure drug (AUC_t_ ∼17.38 ngh/ml). This data revealed that the complex had 2.52 times higher bioavailability as compared to the pure drug. The t_1/2_ (8.34 h) was prolonged (1.89 fold more) in case of the complex as compared with pure drug (4.41 h). Also, the K_e_ was 0.152 h^−1^ which was 0.148 h^−1^ in case of the pure drug showing that the absorption time of the drug has been increased. Based upon these observations, it can be inferred that the bioavailability of the drug was increased with the inclusion complexation.

### Using spray dried complex

Spray dried complexes were orally administered (single oral dose of 10 mg/kg) in rats in order to study its effect on the bioavailability of the drug in comparison to pure drug. The plasma levels were analyzed for the pure ETD over the next 24 h period. Fig. 4c shows the mean serum concentration vs time profile for the spray dried complex indicating sharp increase in plasma levels after oral administration. C_max_ for the complex reached at 3 h post dose was found to be 7.01 ng/ml (2.51 times higher) as compared to pure drug. The t_max_ of the complex was almost similar to that of the pure drug but the extent of absorption was much higher.

Furthermore, AUC_t_ of the complex was found to be ∼31.099 ngh/ml as compared to the pure drug AUC_t_∼17.38 ngh/ml which revealed greater absorption (1.79 times more) of spray dried complex than pure drug. The t_1/2_ (20.76 h) was prolonged (4.71 times) in case of the complex when compared with that of the pure drug (4.41 h). Also, the k_e_ was decreased (0.04 h^−1^) which was 0.15 h^−1^ in case of the pure drug showing that the absorption time of the drug has been increased.

**Fig. 5.**
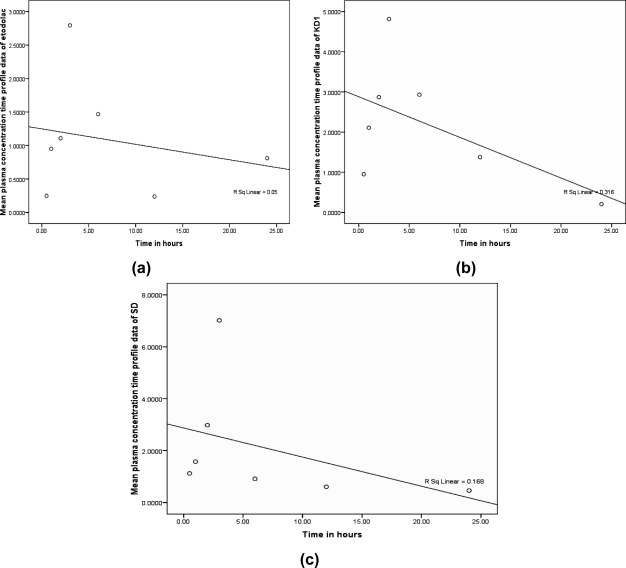
Correlation of mean plasma concentration (AUC_0–24h_) vs time at different intervals for pure drug as well as inclusion complexes (a) ETD (b) KD1 and (c) SD

The curve fitting using least square technique models were used to evaluate the correlation between the mean plasma concentration (AUC_0–24h_) vs time profile of complexes against time in comparison with pure drug as shown in the Fig. 5.

The mean plasma concentration (AUC_0–24h_)-time profile was subjected to curve fitting models using least square method to evaluate the correlation between pure drug or complexes at different time. The r^2^ value of 0.05 for ETD, 0.316 for KD1 (p<0.05) and 0.168 for SD (p<0.05) were obtained form the profile data.

## Experimental

### Materials

ETD was obtained as gift sample from Ranbaxy Pvt. Ltd., (Gurgaon, India) and β-CD was obtained from S.A Chemicals (Mumbai, India). Freshly prepared tripled distilled water was used throughout the study. All the chemicals and solvents used were of analytical grade.

### Preparation of binary inclusion complexes

Kneading and Spray drying techniques were used for the preparation of inclusion complexes. The physiochemical characterization and *in-vitro* dissolution of the inclusion complexes have been well testified in our earlier study [8]. Physical mixtures of ETD and in a molar ratio of 1:1 (PM1) with β–CD was prepared by passing the drug and β–CD through mesh #60 separately and then mixing both solids by simple blending.

### Pharmacodynamic Studies

All the pharmacodynamic studies were carried out on normal LACA mice in accordance with the guidelines of Institutional Animal Care and Use Committee (IACUC). All animals were acclimatized to the laboratory environment before use and were fed on standard normal pellet diet (Aashirwad Industries, Chandigarh) and water *ad libitum.*

### Measurement of Analgesic Activity

#### Tail Flick Method

Tail flick technique was followed to observe the analgesic parameters with the help of Analgesiometer (Popular India Ltd.). Animals (mice) were weighed and basal reaction time of radiant heat was recorded by placing the tip of the tail on radiant heat source. The tail withdrawal from the heat (flicking response) was taken as the end point. A cut off period of 12 seconds was observed to prevent damage to the tail. Six male LACA mice (each of weight 18–25g) were divided into four groups. Group I, group II, group III and group IV received pure drug, KD1, KD2 and SD respectively. Each group received the respective formulation (in a dose equivalent to 10 mg/kg of drug) orally. Percent analgesic effect was measured as follows:

Eq. 1. $$\%\;\text{Analgesic}\;\text{Effect}=\frac{\text{Observed}\;\text{Response}-\text{Baseline}\;\text{value}}{\text{Cutoff}\;\text{value}-\text{Baseline}\;\text{value}}$$

#### Acetic Acid Induced Writhing

Another animal model followed for evaluation of analgesic activity was abdominal writhing assay. Six normal LACA mice (weighing 18–25 g) were divided into five groups each. Group I, group II, group III, group IV and group V received pure drug, PM1, KD1, KD2 and SD respectively. Each group received the respective formulation (in a dose equivalent to 10 mg/kg of drug) orally. Writhing response was elicited by intraperitoneal (i.p.) injection of freshly prepared acetic acid solution (0.6%, 10 ml/kg, i.p.). The number of writhes (constriction of abdomen, turning of trunk, and extension of hind limbs) due to acetic acid was expressed as a nociceptive response. The number of writhes per animal was counted during a 30 min. period, beginning 3 min. after the injection of acetic acid. Percent analgesic effect was measured as follows:

Eq. 2. $$\%\;\text{Analgesic}\;\text{Effect}=\frac{\text{Response}\;\text{for}\;\text{control}-\text{Response}\;\text{for}\;\text{complex}}{\text{Response}\;\text{for}\;\text{complex}}$$

### Evaluation of Anti-Inflammatory Response

#### Carrageenan Induced Rat Paw Edema

Ugo Basile plethysmometer was used to measure the paw volume as an indication of the inflammation. Animals were weighed, numbered and marked on both the hind paws just beyond tibio-tarsal junction, so that every time the paw was dipped in the surfactant column up to the fixed mark to ensure constant paw volume. Initial paw volume (both right and left) of each rat was noted by volume displacement method.

Six animals were divided into six groups: control, pure drug treated, KD1, KD2, SD and PM. Group I, II, III, IV and V received pure drug, PM1, KD1, KD2 and SD respectively. Further, each group received the respective formulation (in a dose equivalent to 10 mg/kg of drug) orally. All the calculations were done with respect to control group. After 30 minutes, 0.1 ml of 1% w/v carrageenan was injected in the plantar region of the left paw of the control group as well as all the groups. The right paw served as a reference non-inflamed paw for comparison. Paw volume of both the legs of three groups and control were measured at 1 h, 2 h, 3 h, 4 h and 6 h after carrageenan challenge. Percent edema inhibition by the test substance was determined as follows:

Eq. 3. $$\%\;\text{Inhibition}\;\text{of}\;\text{inflammation}=\frac{\text{Control}-\text{Test}}{\text{Control}}\cdot 100$$

### Antiulcer activity

The antiulcer activity of the complexes formed was evaluated by testing the complexes for their therapeutic uclerogenic index in mice. Six animals divided into six groups were kept on fasted state for 18 h. Group I was kept as control while groups (II, III, IV, V, and VI) received pure drug, PM1, KD1, KD2 and SD respectively. Control group received 0.5% carboxymethyl cellulose (CMC) while, other groups received the respective formulation (in a dose equivalent to 10 mg/kg of drug) orally. Animals were sacrificed 7 h after the treatment. The stomach was removed, opened along the greater curvature, washed with saline, and observed for ulcers. The ulcers were scored on the following uclerogenic index scale [9].

### HPLC method for estimation of ETD in plasma

100 mg of drug was dissolved in 100 ml of hydro-alcoholic solution. 10 ml of this solution (1 mg/ml) was diluted to 100 ml with triple distilled water to make stock solution of 100 μg/ml. The standards for calibration curve were prepared by adding known amount of ETD to plasma in the range of 100 ng-40 μg/ml. The method for the drug estimation in plasma was validated by RP-HPLC system (LC-10 AT VP Shimadzu pump equipped with a SPD-10 AV P Shimadzu UV- visible detector) consisted of a mobile phase with composition of Acetonitrile: buffer (pH 4.75) (in the ratio of 45:55), at a flow rate of 1.0 mL/min, a C-18 reverse phase column (YMC HPLC column, YMC Co. Ltd. Japan, 250 x 4.6 mm, S-5μ), an injection volume of 20 μL and ultraviolet (UV) detection at wavelength of 225nm.

### Pharmacokinetic Studies

Pharmacokinetic studies were performed on Wistar male rats (by administering pure drug as well as the complexes orally) in order to determine the plasma level profiles of pure ETD and inclusion complexes. The pharmacokinetic profiles of pure drug and inclusion complexes administered orally were statistically compared to evaluate the therapeutic efficacy and in-vivo bioavailability of the complexes. Animals were handled and housed in accordance with the guidelines of IACUC. Five Wistar male rats were divided into three groups consisting of pure drug treated (group I), KD1 (group II) and SD (group III). Each group received the respective formulation (in a dose equivalent to 10 mg/kg of drug) orally. After oral dose, blood samples were withdrawn at 0, 0.5, 1, 2, 3, 6, 12 and 24 h intervals. Blood samples were collected in Ependroff tubes containing heparin. Immediately upon collection, blood samples were centrifuged (5000 rpm for 5 min) and separated plasma was kept at −20°C if not analyzed immediately. They were subjected further for HPLC analysis.

### Statistical Analysis and Curve Fitting

Plasma concentration time profiles were evaluated by fitting these to suitable compartmental approach. Various pharmacokinetic parameters (t_1/2,_ C_max_, T_max ,_ AUC, t_1/2a,_ K_el_ and K_a_) were evaluated using PC-NONLIN software. The pharmacokinetic parameters obtained were evaluated for the significance at 95% level determined by applying one-way ANOVA followed by Tukey’s studentized t-test.

SPSS^®^ (version 16.0) curve-fitting program using least square technique was used to evaluate the correlation between the mean plasma concentration(AUC_0–24h_) against different time intervals and compare their relative goodness of fit for models where a single dependent variable is predicted by a single independent variable or by a time variable as shown in Fig. 5.

## Conclusion

A significant difference in the percentage analgesic response of the inclusion complexes as compared to that of the pure drug was found. The in vivo pharmacokinetic studies after the oral administration of the inclusion complexes showed an enhanced extent of absorption in comparison to the pure drug and the physical mixtures. An increase in the t_1/2_ and decrease in the elimination rate supported the fact that the drug was available for the absorption for a longer time. From these observations, it can be concluded that inclusion complexion phenomena led to enhanced bioavailability (KD1 caused 2.52 times whereas SD increased 1.79 times) than that of pure drug. The order of augmentation of bioavailability was KD1 > SD >PM1 the bioavailability of the drug was increased with the spray dried inclusion complexation. The host–guest strategy that uses β-CD and ETD was found to be very effective and could be successfully employed in the preparation of pharmaceutical formulation of anti-arthritics and analgesics.

## Figures and Tables

**Tab.1.** Anti-ulcer activity of various formulations in rats (n=5, dose eqv. ∼10 mg/kg of drug)

**Group**	**Ulcer Index^a^**
CMC (Control)	0.5 ± 1.02
ETD	5.0 ± 2.11
PM1	2.0 ± 2.17
KD1	1.0 ± 1.23
KD2	1.0 ± 1.44
SD	1.5 ± 1.09

a Data represent mean ± S.D.

**Tab. 2.** Statistical comparison of various pharmacokinetic parameters of ETD, SD, KD1(mean± S.D)^a^

**Subject**	**T_max_ (h)**	**C_max_ (ng/ml)**	**AUC_t_ (ng×h/ml)**	**AUC_∞_ (ng×h/ml)**	**K_a_ (h^−1^)**	**K_el_ (h^−1^)**	**T_½_ (h)**
Etodolac	3.0 ± 0.0	2.795 ± 0.35	17.383 ± 1.22	17.936 ± 1.20	0.309 ± 0.028	0.148 ± 0.011	4.41 ± 0.85
SD	3.0 ± 0.0	7.014 ± 2.97*	31.099 ± 6.93*	44.971 ± 4.54*	0.379 ± 0.020*	0.035 ± 0.009*	20.768 ± 4.87*
KD1	3.0 ± 0.0	4.866 ± 0.07*	43.846 ± 6.5* #	45.537 ± 4.94*	0.441 ± 0.167	0.152 ± 0.04*#	8.34 ± 6.90*#

a Data represent mean ± S.D. (standard deviation);

* Significantly different from group I at 95% level determined by applying one-way ANOVA followed by Tukey’s studentized t-test;

# Significantly different from group SD at 95% level determined by applying one way ANOVA followed by Tukey’s studentized t-test.

## Abbreviations

KD: Kneading complex (KD1 (1:1) or KD2 (1:2) denotes ratio of complextation with β-CD)
SD: Spray dried complex
PM1: Physical mixture
ETD: Etodolac
